# *cis*-Dichloridobis{[4-(dimethyl­amino)­phen­yl]diphenyl­phosphane-κ*P*}platinum(II) ethyl acetate monosolvate

**DOI:** 10.1107/S1600536811035628

**Published:** 2011-09-14

**Authors:** Alfred Muller, Reinout Meijboom

**Affiliations:** a Research Centre for Synthesis and Catalysis, Department of Chemistry, University of Johannesburg, PO Box 524, Auckland Park, 2006 Johannesburg, South Africa

## Abstract

The title compound, [PtCl_2_(C_20_H_20_P)_2_]·C_4_H_8_O_2_, crystallizes with the Pt atom in a distorted *cis*-square-planar geometry. The Pt—P bond lengths are 2.2490 (19) and 2.253 (2) Å, and the Pt—Cl bond lengths are 2.344 (2) and 2.3475 (18) Å. Some weak C—H⋯Cl and C—H⋯O inter­actions involving the solvate mol­ecule were observed.

## Related literature

For a review on related compounds, see: Spessard & Miessler (1996 ▶). For the synthesis of the starting materials, see: Drew & Doyle (1990 ▶).
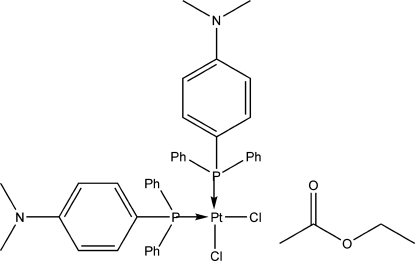


## Experimental

### 

#### Crystal data

[PtCl_2_(C_20_H_20_P)_2_]·C_4_H_8_O_2_
*M* *_r_* = 964.76
Monoclinic, 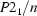

*a* = 11.8148 (7) Å
*b* = 19.1072 (11) Å
*c* = 18.5668 (13) Å
β = 104.732 (4)°
*V* = 4053.6 (4) Å^3^
*Z* = 4
Mo *K*α radiation
μ = 3.71 mm^−1^
*T* = 100 K
0.14 × 0.08 × 0.04 mm

#### Data collection

Bruker X8 APEXII 4K KappaCCD diffractometer
Absorption correction: multi-scan (*SADABS*; Bruker, 2004 ▶) *T*_min_ = 0.625, *T*_max_ = 0.866
47227 measured reflections
10080 independent reflections
6044 reflections with *I* > 2σ(*I*)
*R*_int_ = 0.143

#### Refinement

*R*[*F* ^2^ > 2σ(*F* ^2^)] = 0.058
*wR*(*F* ^2^) = 0.144
*S* = 1.01
10080 reflections
484 parameters
6 restraints
H-atom parameters constrained
Δρ_max_ = 1.53 e Å^−3^
Δρ_min_ = −1.87 e Å^−3^

### 

Data collection: *APEX2* (Bruker, 2005 ▶); cell refinement: *SAINT-Plus* (Bruker, 2004 ▶); data reduction: *SAINT-Plus* and *XPREP* (Bruker, 2004 ▶); program(s) used to solve structure: *SIR97* (Altomare *et al.*, 1999 ▶); program(s) used to refine structure: *SHELXL97* (Sheldrick, 2008 ▶); molecular graphics: *DIAMOND* (Brandenburg & Putz, 2005 ▶); software used to prepare material for publication: *WinGX* (Farrugia, 1999 ▶).

## Supplementary Material

Crystal structure: contains datablock(s) global, I. DOI: 10.1107/S1600536811035628/rk2293sup1.cif

Structure factors: contains datablock(s) I. DOI: 10.1107/S1600536811035628/rk2293Isup2.hkl

Additional supplementary materials:  crystallographic information; 3D view; checkCIF report

## Figures and Tables

**Table 1:** Hydrogen-bond geometry (Å, °)

*D*—H⋯*A*	*D*—H	H⋯*A*	*D*⋯*A*	*D*—H⋯*A*
C5—H5*B*⋯Cl2^i^	0.98	2.79	3.601 (10)	141
C64—H64⋯O1^ii^	0.95	2.39	3.317 (11)	166

Symmetry codes: (i) 
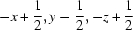
; (ii) 
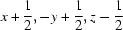
.

**Fig. 1.**
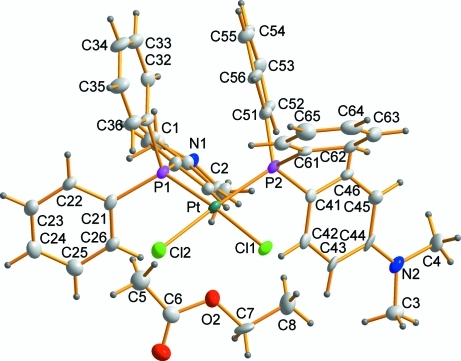
The structure of title compound with the atom numbering scheme. Displacement ellipsoids are drawn at 50% probability level. H atoms are presented as a small spheres of arbitrary radius. For the C atoms, the first digit indicates ring number and the second digit indicates the position of the atom in the ring. Some lables have been omitted for clarity, all rings have been numbered in the same, systematic manner.

## supplementary crystallographic information

### Comment

Transition metal complexes containing phosphine, arsine and stibine ligands are widely being investigated in various fields of organometallic chemistry (Spessard & Miessler, 1996). As part of a systematic investigation involving complexes with the general formula *trans*–[*MX*_2_(*L*)_2_] (*M* = Pt or Pd; *X* = halogen, *Me*, *Ph*; *L* = Group 15 donor ligand), crystals of the title compound were obtained.

The PtCl_2_(*L*)_2_ (*L* = tertiary phosphine, arsine or stibine) complexes can conveniently be prepared by the substitution of 1,5–cyclooctadiene (*COD*) from [PtCl_2_(*COD*)]. The title compound, *cis*–{PtCl_2_[P*Ph*_2_(4–*Me*_2_NC_6_H_4_)]_2_}, crystallizes in the monoclinic space group *P*2_1_/*n*, with each pair of equivalent ligands in a mutually *cis*–orientation. The geometry is slightly distorted square planar and the Pt atom is not elevated out of the coordinating atom plane. All angles in the coordination polyhedron are close to the ideal value of 90°, with P1—Pt—P2 = 98.43 (7)° and Cl1—Pt—Cl2 = 87.13 (7)°. The Cl1—Pt—P angles are 175.95 (7)° and 84.93 (7)° respectively for P1 and P2. Some weak intermolecular interactions were observed and are reported in Table 1.

The title compound compares well with other closely related Pt(II) complexes from the literature containing two chloro and two tertiary phosphine ligands in a *cis*–geometry. The title compound, having Pt—Cl bond lengths of 2.344 (2)Å and 2.3475 (18)Å and Pd—P bond lengths of 2.2490 (19)Å and 2.253 (2)Å, fits well into the typical range for complexes of this kind. It is notable that the title compound crystallized as a solvated complex, as these type of Pt(II) complexes tend to crystallize as solvates.

### Experimental

Dichloro(1,5–cyclooctadiene)platinum(II), PtCl_2_(*COD*), was prepared according to the literature procedure of Drew & Doyle (1990). A solution of diphenyl(4–dimethylaminophenyl)phosphine (0.2 mmol) in ethyl acetate (2.0 cm^3^) was added to a solution of PdCl_2_(*COD*) (0.1 mmol) in dichloromethane (3.0 cm^3^). Recrystallization from ethyl acetate gave light yellow crystals of the title compound.

### Refinement

The aromatic, methylene, and methyl H atoms were placed in geometrically idealized positions (C—H = 0.95Å–0.98Å) and constrained to ride on their parent atoms with *U*_iso_(H) = 1.2*U*_eq_(C) for aromatic and methylene H atoms, and *U*_iso_(H) = 1.5*U*_eq_(C) for methyl H atoms respectively. Methyl torsion angles were refined from electron density.

The highest residual electron density peak of 1.53 e×Å^3^ is 1.14Å from Pt and the deepest hole of -1.87 e×Å^3^ is 0.87Å from Pt representing no physical meaning.

### Crystal data

**Table:**

[PtCl_2_(C_20_H_20_P)_2_]·C_4_H_8_O_2_	*F*(000) = 1936
*M**_r_* = 964.76	*D*_x_ = 1.581 Mg m^−^^3^
Monoclinic, *P*2_1_/*n*	Mo *K*α radiation, λ = 0.71073 Å
Hall symbol: -P 2yn	Cell parameters from 3702 reflections
*a* = 11.8148 (7) Å	θ = 2.5–23.6°
*b* = 19.1072 (11) Å	µ = 3.71 mm^−^^1^
*c* = 18.5668 (13) Å	*T* = 100 K
β = 104.732 (4)°	Prism, light yellow
*V* = 4053.6 (4) Å^3^	0.14 × 0.08 × 0.04 mm
*Z* = 4	

### Data collection

**Table:**

Bruker X8 APEXII 4K KappaCCD diffractometer	6044 reflections with *I* > 2σ(*I*)
Graphite	*R*_int_ = 0.143
φ and ω scans	θ_max_ = 28.3°, θ_min_ = 1.6°
Absorption correction: multi-scan (*SADABS*; Bruker, 2004)	*h* = −15→15
*T*_min_ = 0.625, *T*_max_ = 0.866	*k* = −25→23
47227 measured reflections	*l* = −24→24
10080 independent reflections	

### Refinement

**Table:**

Refinement on *F*^2^	Primary atom site location: structure-invariant direct methods
Least-squares matrix: full	Secondary atom site location: difference Fourier map
*R*[*F*^2^ > 2σ(*F*^2^)] = 0.058	Hydrogen site location: inferred from neighbouring sites
*wR*(*F*^2^) = 0.144	H-atom parameters constrained
*S* = 1.01	*w* = 1/[σ^2^(*F*_o_^2^) + (0.0541*P*)^2^] where *P* = (*F*_o_^2^ + 2*F*_c_^2^)/3
10080 reflections	(Δ/σ)_max_ = 0.001
484 parameters	Δρ_max_ = 1.53 e Å^−^^3^
6 restraints	Δρ_min_ = −1.87 e Å^−^^3^

### Special details

**Table:**

Geometry. All s.u.'s (except the s.u. in the dihedral angle between two l.s. planes) are estimated using the full covariance matrix. The cell s.u.'s are taken into account individually in the estimation of s.u.'s in distances, angles and torsion angles; correlations between s.u.'s in cell parameters are only used when they are defined by crystal symmetry. An approximate (isotropic) treatment of cell s.u.'s is used for estimating s.u.'s involving l.s. planes.
Refinement. Refinement of *F*^2^ against ALL reflections. The weighted *R*–factor *wR* and goodness of fit *S* are based on *F*^2^, conventional *R*–factors *R* are based on *F*, with *F* set to zero for negative *F*^2^. The threshold expression of *F*^2^ > σ(*F*^2^) is used only for calculating *R*–factors(gt) *etc*. and is not relevant to the choice of reflections for refinement. *R*–factors based on *F*^2^ are statistically about twice as large as those based on *F*, and *R*–factors based on ALL data will be even larger.

### Fractional atomic coordinates and isotropic or equivalent isotropic displacement parameters (Å^2^)

**Table:**

	*x*	*y*	*z*	*U*_iso_*/*U*_eq_	
Pt	0.41608 (2)	0.272508 (17)	0.144139 (17)	0.01765 (10)	
P1	0.24750 (16)	0.21421 (11)	0.09926 (11)	0.0182 (5)	
P2	0.52011 (15)	0.22582 (12)	0.06977 (11)	0.0189 (4)	
Cl1	0.58930 (15)	0.33308 (11)	0.19929 (11)	0.0232 (5)	
Cl2	0.32651 (16)	0.33794 (12)	0.22107 (12)	0.0283 (5)	
C11	0.2574 (6)	0.1215 (5)	0.0909 (4)	0.0226 (19)	
C12	0.1588 (6)	0.0804 (4)	0.0589 (4)	0.0224 (19)	
H12	0.0876	0.1031	0.035	0.027*	
C13	0.1616 (6)	0.0082 (4)	0.0610 (4)	0.0202 (18)	
H13	0.094	−0.0175	0.0364	0.024*	
C14	0.2645 (6)	−0.0284 (4)	0.0994 (4)	0.0183 (17)	
C15	0.3618 (6)	0.0134 (4)	0.1327 (4)	0.0176 (17)	
H15	0.433	−0.0087	0.1577	0.021*	
C16	0.3566 (6)	0.0842 (5)	0.1301 (4)	0.0209 (18)	
H16	0.4233	0.1099	0.156	0.025*	
C1	0.1571 (6)	−0.1382 (5)	0.0803 (5)	0.0250 (19)	
H1A	0.1038	−0.1223	0.1096	0.038*	
H1B	0.1734	−0.1882	0.0892	0.038*	
H1C	0.1208	−0.1305	0.0272	0.038*	
C2	0.3666 (7)	−0.1350 (5)	0.1502 (5)	0.027 (2)	
H2A	0.4388	−0.1151	0.1422	0.04*	
H2B	0.363	−0.185	0.1382	0.04*	
H2C	0.3655	−0.1287	0.2024	0.04*	
N1	0.2652 (5)	−0.0993 (4)	0.1019 (4)	0.0229 (16)	
C21	0.1454 (6)	0.2173 (4)	0.1589 (4)	0.0185 (17)	
C22	0.0286 (6)	0.2383 (4)	0.1329 (4)	0.0229 (19)	
H22	−0.0001	0.2549	0.0834	0.028*	
C23	−0.0448 (7)	0.2344 (5)	0.1806 (5)	0.028 (2)	
H23	−0.1239	0.2492	0.1639	0.034*	
C24	−0.0035 (7)	0.2094 (5)	0.2519 (5)	0.027 (2)	
H24	−0.0549	0.2053	0.2836	0.033*	
C25	0.1119 (7)	0.1903 (5)	0.2774 (5)	0.026 (2)	
H25	0.1409	0.1739	0.327	0.031*	
C26	0.1852 (6)	0.1950 (4)	0.2310 (4)	0.0226 (19)	
H26	0.2652	0.1824	0.2492	0.027*	
C31	0.1652 (6)	0.2525 (4)	0.0108 (4)	0.0207 (18)	
C32	0.1250 (6)	0.2170 (4)	−0.0550 (4)	0.0217 (18)	
H32	0.1399	0.1683	−0.057	0.026*	
C33	0.0625 (7)	0.2515 (5)	−0.1188 (5)	0.026 (2)	
H33	0.0364	0.2264	−0.1642	0.031*	
C34	0.0387 (7)	0.3208 (5)	−0.1163 (5)	0.034 (2)	
H34	−0.0058	0.3441	−0.1595	0.041*	
C35	0.0799 (7)	0.3575 (5)	−0.0503 (5)	0.032 (2)	
H35	0.0642	0.4061	−0.0488	0.038*	
C36	0.1430 (7)	0.3243 (5)	0.0127 (5)	0.031 (2)	
H36	0.1715	0.3499	0.0575	0.037*	
C41	0.6309 (6)	0.1657 (4)	0.1169 (4)	0.0175 (17)	
C42	0.6565 (6)	0.1574 (4)	0.1945 (4)	0.0206 (18)	
H42	0.6152	0.1849	0.222	0.025*	
C43	0.7400 (6)	0.1105 (4)	0.2322 (5)	0.0224 (19)	
H43	0.7527	0.1048	0.2844	0.027*	
C44	0.8064 (6)	0.0711 (4)	0.1933 (5)	0.0222 (19)	
C45	0.7817 (6)	0.0793 (5)	0.1154 (4)	0.0231 (19)	
H45	0.8246	0.0529	0.0879	0.028*	
C46	0.6956 (6)	0.1252 (4)	0.0781 (4)	0.0199 (18)	
H46	0.6800	0.1295	0.0256	0.024*	
N2	0.8877 (5)	0.0230 (4)	0.2294 (4)	0.0260 (17)	
C3	0.9274 (7)	0.0223 (5)	0.3106 (5)	0.029 (2)	
H3A	0.9657	0.0669	0.3281	0.044*	
H3B	0.9831	−0.0161	0.3266	0.044*	
H3C	0.8601	0.0156	0.3317	0.044*	
C4	0.9620 (7)	−0.0137 (5)	0.1886 (5)	0.032 (2)	
H4A	0.9124	−0.036	0.1442	0.048*	
H4B	1.0085	−0.0495	0.2209	0.048*	
H4C	1.0144	0.02	0.1738	0.048*	
C51	0.4439 (6)	0.1813 (5)	−0.0155 (4)	0.0204 (18)	
C52	0.4352 (6)	0.1089 (5)	−0.0196 (4)	0.0226 (19)	
H52	0.4735	0.0814	0.0221	0.027*	
C53	0.3707 (6)	0.0758 (5)	−0.0845 (5)	0.027 (2)	
H53	0.365	0.0263	−0.0869	0.033*	
C54	0.3155 (6)	0.1164 (5)	−0.1449 (5)	0.030 (2)	
H54	0.2701	0.0944	−0.1886	0.037*	
C55	0.3254 (6)	0.1879 (5)	−0.1425 (5)	0.0240 (19)	
H55	0.2881	0.2152	−0.1847	0.029*	
C56	0.3899 (6)	0.2203 (5)	−0.0782 (4)	0.0222 (18)	
H56	0.3974	0.2698	−0.077	0.027*	
C61	0.5940 (6)	0.2958 (4)	0.0322 (4)	0.0171 (17)	
C62	0.7032 (6)	0.2860 (4)	0.0172 (4)	0.0210 (19)	
H62	0.7409	0.2418	0.0268	0.025*	
C63	0.7571 (7)	0.3399 (5)	−0.0116 (5)	0.033 (2)	
H63	0.8305	0.3324	−0.0225	0.04*	
C64	0.7032 (8)	0.4048 (5)	−0.0245 (5)	0.034 (2)	
H64	0.7407	0.4422	−0.043	0.041*	
C65	0.5950 (8)	0.4152 (5)	−0.0103 (5)	0.036 (2)	
H65	0.5583	0.4597	−0.0195	0.043*	
C66	0.5398 (7)	0.3613 (5)	0.0173 (5)	0.027 (2)	
H66	0.4649	0.3688	0.0261	0.033*	
O1	0.3757 (5)	−0.0196 (4)	0.4216 (3)	0.0376 (16)	
O2	0.4524 (5)	−0.0288 (3)	0.3231 (3)	0.0317 (15)	
C5	0.2629 (7)	0.0185 (5)	0.3013 (5)	0.038 (2)	
H5A	0.212	0.0418	0.3282	0.057*	
H5B	0.2197	−0.0195	0.2708	0.057*	
H5C	0.2882	0.0525	0.2691	0.057*	
C6	0.3671 (7)	−0.0107 (5)	0.3557 (5)	0.026 (2)	
C7	0.5553 (7)	−0.0609 (5)	0.3705 (5)	0.035 (2)	
H7A	0.5341	−0.1049	0.3921	0.042*	
H7B	0.5925	−0.029	0.4117	0.042*	
C8	0.6377 (7)	−0.0758 (5)	0.3222 (6)	0.040 (3)	
H8A	0.6001	−0.1079	0.2821	0.06*	
H8B	0.7095	−0.0971	0.3525	0.06*	
H8C	0.6569	−0.0319	0.3007	0.06*	

### Atomic displacement parameters (Å^2^)

**Table:**

	*U*^11^	*U*^22^	*U*^33^	*U*^12^	*U*^13^	*U*^23^
Pt	0.01160 (13)	0.02100 (19)	0.01731 (15)	−0.00091 (14)	−0.00192 (10)	−0.00105 (16)
P1	0.0129 (9)	0.0214 (13)	0.0183 (10)	0.0001 (8)	0.0004 (8)	0.0029 (9)
P2	0.0118 (8)	0.0226 (12)	0.0191 (10)	−0.0001 (9)	−0.0016 (7)	0.0012 (10)
Cl1	0.0141 (8)	0.0287 (13)	0.0231 (10)	−0.0062 (8)	−0.0016 (7)	−0.0039 (9)
Cl2	0.0202 (9)	0.0334 (14)	0.0301 (11)	−0.0041 (9)	0.0044 (8)	−0.0120 (10)
C11	0.016 (4)	0.026 (5)	0.024 (4)	0.003 (3)	0.003 (3)	−0.002 (4)
C12	0.012 (4)	0.026 (5)	0.026 (4)	0.007 (3)	−0.001 (3)	0.000 (4)
C13	0.017 (4)	0.021 (5)	0.021 (4)	0.003 (3)	0.001 (3)	−0.003 (4)
C14	0.019 (4)	0.015 (5)	0.022 (4)	0.000 (3)	0.005 (3)	0.000 (4)
C15	0.010 (3)	0.018 (5)	0.020 (4)	0.001 (3)	−0.005 (3)	0.003 (3)
C16	0.016 (4)	0.030 (5)	0.017 (4)	0.002 (3)	0.003 (3)	−0.001 (4)
C1	0.019 (4)	0.028 (5)	0.027 (5)	−0.003 (4)	0.004 (3)	−0.002 (4)
C2	0.021 (4)	0.021 (5)	0.035 (5)	0.003 (4)	0.000 (4)	−0.001 (4)
N1	0.015 (3)	0.023 (4)	0.028 (4)	−0.003 (3)	0.001 (3)	−0.002 (3)
C21	0.014 (3)	0.013 (5)	0.026 (4)	−0.001 (3)	0.001 (3)	−0.001 (4)
C22	0.015 (3)	0.029 (6)	0.021 (4)	0.002 (3)	−0.002 (3)	0.001 (4)
C23	0.020 (4)	0.035 (6)	0.027 (5)	0.003 (4)	0.003 (3)	−0.004 (4)
C24	0.024 (4)	0.027 (6)	0.034 (5)	−0.008 (4)	0.012 (4)	−0.003 (4)
C25	0.031 (4)	0.020 (5)	0.028 (5)	0.001 (4)	0.009 (4)	−0.001 (4)
C26	0.016 (4)	0.025 (5)	0.024 (4)	0.002 (3)	−0.001 (3)	−0.002 (4)
C31	0.016 (4)	0.026 (5)	0.017 (4)	−0.003 (3)	0.000 (3)	0.007 (3)
C32	0.020 (4)	0.019 (5)	0.027 (4)	−0.008 (3)	0.007 (3)	−0.003 (4)
C33	0.023 (4)	0.027 (5)	0.024 (4)	−0.005 (3)	−0.001 (3)	0.000 (4)
C34	0.028 (4)	0.042 (7)	0.025 (5)	0.002 (4)	−0.008 (4)	0.006 (5)
C35	0.034 (5)	0.030 (6)	0.027 (5)	0.008 (4)	−0.003 (4)	0.002 (4)
C36	0.025 (4)	0.037 (6)	0.026 (5)	0.001 (4)	−0.001 (4)	0.001 (4)
C41	0.011 (3)	0.021 (5)	0.020 (4)	−0.005 (3)	0.004 (3)	0.001 (3)
C42	0.009 (3)	0.024 (5)	0.028 (4)	−0.004 (3)	0.003 (3)	0.002 (4)
C43	0.007 (3)	0.031 (5)	0.027 (4)	−0.001 (3)	−0.002 (3)	0.002 (4)
C44	0.007 (3)	0.023 (5)	0.032 (4)	−0.002 (3)	−0.004 (3)	−0.003 (4)
C45	0.020 (4)	0.025 (5)	0.023 (4)	0.000 (3)	0.003 (3)	−0.003 (4)
C46	0.014 (3)	0.028 (5)	0.015 (4)	0.000 (3)	−0.002 (3)	0.003 (4)
N2	0.016 (3)	0.028 (5)	0.030 (4)	0.001 (3)	−0.001 (3)	0.004 (3)
C3	0.030 (4)	0.030 (6)	0.024 (5)	0.002 (4)	0.002 (4)	0.005 (4)
C4	0.020 (4)	0.036 (6)	0.036 (5)	0.014 (4)	0.003 (4)	0.003 (5)
C51	0.015 (4)	0.026 (5)	0.019 (4)	0.004 (3)	0.001 (3)	−0.001 (4)
C52	0.016 (4)	0.028 (6)	0.020 (4)	0.001 (3)	−0.002 (3)	0.001 (4)
C53	0.016 (4)	0.034 (6)	0.030 (5)	−0.010 (4)	0.003 (4)	−0.005 (4)
C54	0.014 (4)	0.055 (7)	0.018 (4)	−0.005 (4)	−0.002 (3)	−0.009 (4)
C55	0.022 (4)	0.022 (5)	0.025 (4)	−0.001 (4)	−0.001 (3)	−0.001 (4)
C56	0.016 (3)	0.024 (5)	0.022 (4)	−0.003 (3)	−0.002 (3)	−0.002 (4)
C61	0.018 (4)	0.021 (5)	0.010 (3)	−0.001 (3)	−0.001 (3)	0.004 (3)
C62	0.017 (4)	0.023 (5)	0.019 (4)	−0.001 (3)	−0.004 (3)	0.002 (4)
C63	0.020 (4)	0.054 (7)	0.024 (5)	−0.007 (4)	0.003 (4)	0.001 (5)
C64	0.042 (5)	0.034 (6)	0.023 (5)	−0.016 (5)	0.003 (4)	−0.003 (4)
C65	0.041 (5)	0.034 (6)	0.031 (5)	0.001 (5)	0.007 (4)	0.000 (5)
C66	0.025 (4)	0.033 (6)	0.022 (4)	−0.001 (4)	0.001 (4)	0.004 (4)
O1	0.040 (4)	0.046 (5)	0.028 (4)	0.008 (3)	0.011 (3)	0.005 (3)
O2	0.023 (3)	0.042 (4)	0.030 (3)	0.008 (3)	0.007 (3)	0.007 (3)
C5	0.037 (5)	0.036 (7)	0.040 (6)	0.005 (4)	0.007 (5)	0.004 (5)
C6	0.030 (4)	0.021 (5)	0.028 (5)	−0.005 (4)	0.010 (4)	−0.001 (4)
C7	0.033 (5)	0.047 (7)	0.016 (4)	0.015 (4)	−0.011 (4)	−0.002 (4)
C8	0.027 (5)	0.042 (7)	0.051 (6)	0.011 (4)	0.010 (5)	0.004 (5)

### Geometric parameters (Å, °)

**Table:**

Pt—P1	2.2490 (19)	C42—C43	1.384 (10)
Pt—P2	2.253 (2)	C42—H42	0.95
Pt—Cl2	2.344 (2)	C43—C44	1.410 (11)
Pt—Cl1	2.3475 (18)	C43—H43	0.95
P1—C11	1.785 (9)	C44—N2	1.374 (10)
P1—C21	1.834 (8)	C44—C45	1.411 (11)
P1—C31	1.835 (8)	C45—C46	1.388 (10)
P2—C41	1.793 (8)	C45—H45	0.95
P2—C51	1.821 (8)	C46—H46	0.95
P2—C61	1.830 (8)	N2—C3	1.460 (10)
C11—C12	1.406 (11)	N2—C4	1.475 (10)
C11—C16	1.406 (10)	C3—H3A	0.98
C12—C13	1.380 (11)	C3—H3B	0.98
C12—H12	0.95	C3—H3C	0.98
C13—C14	1.426 (10)	C4—H4A	0.98
C13—H13	0.95	C4—H4B	0.98
C14—N1	1.354 (10)	C4—H4C	0.98
C14—C15	1.406 (10)	C51—C52	1.388 (12)
C15—C16	1.355 (11)	C51—C56	1.392 (11)
C15—H15	0.95	C52—C53	1.402 (11)
C16—H16	0.95	C52—H52	0.95
C1—N1	1.444 (9)	C53—C54	1.382 (12)
C1—H1A	0.98	C53—H53	0.95
C1—H1B	0.98	C54—C55	1.372 (12)
C1—H1C	0.98	C54—H54	0.95
C2—N1	1.469 (10)	C55—C56	1.388 (10)
C2—H2A	0.98	C55—H55	0.95
C2—H2B	0.98	C56—H56	0.95
C2—H2C	0.98	C61—C62	1.400 (10)
C21—C26	1.369 (11)	C61—C66	1.401 (11)
C21—C22	1.400 (10)	C62—C63	1.388 (12)
C22—C23	1.390 (11)	C62—H62	0.95
C22—H22	0.95	C63—C64	1.385 (13)
C23—C24	1.374 (12)	C63—H63	0.95
C23—H23	0.95	C64—C65	1.384 (12)
C24—C25	1.375 (11)	C64—H64	0.95
C24—H24	0.95	C65—C66	1.385 (12)
C25—C26	1.372 (11)	C65—H65	0.95
C25—H25	0.95	C66—H66	0.95
C26—H26	0.95	O1—C6	1.215 (10)
C31—C32	1.371 (11)	O2—C6	1.346 (9)
C31—C36	1.399 (12)	O2—C7	1.444 (9)
C32—C33	1.392 (11)	C5—C6	1.489 (12)
C32—H32	0.95	C5—H5A	0.98
C33—C34	1.358 (13)	C5—H5B	0.98
C33—H33	0.95	C5—H5C	0.98
C34—C35	1.387 (12)	C7—C8	1.509 (12)
C34—H34	0.95	C7—H7A	0.99
C35—C36	1.373 (11)	C7—H7B	0.99
C35—H35	0.95	C8—H8A	0.98
C36—H36	0.95	C8—H8B	0.98
C41—C42	1.403 (10)	C8—H8C	0.98
C41—C46	1.407 (11)		
			
P1—Pt—P2	98.43 (7)	C46—C41—P2	121.7 (6)
P1—Pt—Cl2	89.74 (7)	C43—C42—C41	122.1 (8)
P2—Pt—Cl2	170.42 (8)	C43—C42—H42	118.9
P1—Pt—Cl1	175.96 (7)	C41—C42—H42	118.9
P2—Pt—Cl1	84.93 (7)	C42—C43—C44	120.3 (8)
Cl2—Pt—Cl1	87.13 (7)	C42—C43—H43	119.8
C11—P1—C21	98.8 (4)	C44—C43—H43	119.8
C11—P1—C31	110.4 (4)	N2—C44—C43	121.2 (8)
C21—P1—C31	104.4 (3)	N2—C44—C45	120.7 (7)
C11—P1—Pt	116.9 (2)	C43—C44—C45	118.0 (7)
C21—P1—Pt	114.6 (3)	C46—C45—C44	121.0 (8)
C31—P1—Pt	110.6 (3)	C46—C45—H45	119.5
C41—P2—C51	105.3 (4)	C44—C45—H45	119.5
C41—P2—C61	107.1 (3)	C45—C46—C41	121.2 (7)
C51—P2—C61	100.8 (4)	C45—C46—H46	119.4
C41—P2—Pt	113.5 (3)	C41—C46—H46	119.4
C51—P2—Pt	119.4 (2)	C44—N2—C3	120.7 (7)
C61—P2—Pt	109.4 (3)	C44—N2—C4	120.1 (7)
C12—C11—C16	115.3 (8)	C3—N2—C4	116.7 (6)
C12—C11—P1	121.7 (6)	N2—C3—H3A	109.5
C16—C11—P1	121.5 (6)	N2—C3—H3B	109.5
C13—C12—C11	122.3 (7)	H3A—C3—H3B	109.5
C13—C12—H12	118.8	N2—C3—H3C	109.5
C11—C12—H12	118.8	H3A—C3—H3C	109.5
C12—C13—C14	121.1 (7)	H3B—C3—H3C	109.5
C12—C13—H13	119.5	N2—C4—H4A	109.5
C14—C13—H13	119.5	N2—C4—H4B	109.5
N1—C14—C15	123.7 (7)	H4A—C4—H4B	109.5
N1—C14—C13	120.3 (7)	N2—C4—H4C	109.5
C15—C14—C13	116.0 (7)	H4A—C4—H4C	109.5
C16—C15—C14	121.8 (7)	H4B—C4—H4C	109.5
C16—C15—H15	119.1	C52—C51—C56	118.4 (7)
C14—C15—H15	119.1	C52—C51—P2	121.7 (6)
C15—C16—C11	123.3 (7)	C56—C51—P2	119.8 (7)
C15—C16—H16	118.3	C51—C52—C53	120.9 (8)
C11—C16—H16	118.3	C51—C52—H52	119.6
N1—C1—H1A	109.5	C53—C52—H52	119.6
N1—C1—H1B	109.5	C54—C53—C52	119.1 (9)
H1A—C1—H1B	109.5	C54—C53—H53	120.5
N1—C1—H1C	109.5	C52—C53—H53	120.5
H1A—C1—H1C	109.5	C55—C54—C53	120.9 (8)
H1B—C1—H1C	109.5	C55—C54—H54	119.6
N1—C2—H2A	109.5	C53—C54—H54	119.6
N1—C2—H2B	109.5	C54—C55—C56	119.7 (8)
H2A—C2—H2B	109.5	C54—C55—H55	120.1
N1—C2—H2C	109.5	C56—C55—H55	120.1
H2A—C2—H2C	109.5	C55—C56—C51	121.0 (8)
H2B—C2—H2C	109.5	C55—C56—H56	119.5
C14—N1—C1	120.5 (6)	C51—C56—H56	119.5
C14—N1—C2	118.8 (6)	C62—C61—C66	118.6 (7)
C1—N1—C2	117.2 (7)	C62—C61—P2	122.1 (6)
C26—C21—C22	119.4 (7)	C66—C61—P2	119.3 (6)
C26—C21—P1	117.7 (5)	C63—C62—C61	120.9 (8)
C22—C21—P1	122.9 (6)	C63—C62—H62	119.6
C23—C22—C21	119.0 (7)	C61—C62—H62	119.6
C23—C22—H22	120.5	C64—C63—C62	119.7 (8)
C21—C22—H22	120.5	C64—C63—H63	120.2
C24—C23—C22	120.4 (7)	C62—C63—H63	120.2
C24—C23—H23	119.8	C65—C64—C63	120.1 (9)
C22—C23—H23	119.8	C65—C64—H64	119.9
C23—C24—C25	120.1 (8)	C63—C64—H64	119.9
C23—C24—H24	119.9	C64—C65—C66	120.6 (9)
C25—C24—H24	119.9	C64—C65—H65	119.7
C26—C25—C24	119.7 (8)	C66—C65—H65	119.7
C26—C25—H25	120.1	C65—C66—C61	120.1 (8)
C24—C25—H25	120.1	C65—C66—H66	119.9
C21—C26—C25	121.3 (7)	C61—C66—H66	119.9
C21—C26—H26	119.3	C6—O2—C7	116.4 (7)
C25—C26—H26	119.3	C6—C5—H5A	109.5
C32—C31—C36	118.9 (7)	C6—C5—H5B	109.5
C32—C31—P1	125.8 (7)	H5A—C5—H5B	109.5
C36—C31—P1	115.3 (6)	C6—C5—H5C	109.5
C31—C32—C33	120.8 (8)	H5A—C5—H5C	109.5
C31—C32—H32	119.6	H5B—C5—H5C	109.5
C33—C32—H32	119.6	O1—C6—O2	122.5 (8)
C34—C33—C32	120.2 (8)	O1—C6—C5	125.4 (8)
C34—C33—H33	119.9	O2—C6—C5	112.1 (7)
C32—C33—H33	119.9	O2—C7—C8	106.7 (7)
C33—C34—C35	119.6 (8)	O2—C7—H7A	110.4
C33—C34—H34	120.2	C8—C7—H7A	110.4
C35—C34—H34	120.2	O2—C7—H7B	110.4
C36—C35—C34	120.8 (9)	C8—C7—H7B	110.4
C36—C35—H35	119.6	H7A—C7—H7B	108.6
C34—C35—H35	119.6	C7—C8—H8A	109.5
C35—C36—C31	119.7 (8)	C7—C8—H8B	109.5
C35—C36—H36	120.2	H8A—C8—H8B	109.5
C31—C36—H36	120.2	C7—C8—H8C	109.5
C42—C41—C46	117.3 (7)	H8A—C8—H8C	109.5
C42—C41—P2	120.9 (6)	H8B—C8—H8C	109.5
			
P2—Pt—P1—C11	49.7 (3)	C33—C34—C35—C36	−0.9 (13)
Cl2—Pt—P1—C11	−135.3 (3)	C34—C35—C36—C31	−0.6 (13)
P2—Pt—P1—C21	164.6 (3)	C32—C31—C36—C35	1.2 (12)
Cl2—Pt—P1—C21	−20.5 (3)	P1—C31—C36—C35	−178.6 (7)
P2—Pt—P1—C31	−77.8 (3)	C51—P2—C41—C42	−140.1 (6)
Cl2—Pt—P1—C31	97.1 (3)	C61—P2—C41—C42	113.2 (6)
P1—Pt—P2—C41	−107.5 (3)	Pt—P2—C41—C42	−7.6 (7)
Cl1—Pt—P2—C41	70.2 (3)	C51—P2—C41—C46	40.2 (7)
P1—Pt—P2—C51	17.6 (3)	C61—P2—C41—C46	−66.6 (7)
Cl1—Pt—P2—C51	−164.6 (3)	Pt—P2—C41—C46	172.6 (5)
P1—Pt—P2—C61	132.9 (2)	C46—C41—C42—C43	−1.4 (11)
Cl1—Pt—P2—C61	−49.3 (2)	P2—C41—C42—C43	178.8 (6)
C21—P1—C11—C12	61.0 (7)	C41—C42—C43—C44	2.7 (12)
C31—P1—C11—C12	−48.0 (8)	C42—C43—C44—N2	−178.4 (7)
Pt—P1—C11—C12	−175.6 (6)	C42—C43—C44—C45	−2.3 (11)
C21—P1—C11—C16	−104.7 (7)	N2—C44—C45—C46	176.8 (7)
C31—P1—C11—C16	146.3 (6)	C43—C44—C45—C46	0.7 (12)
Pt—P1—C11—C16	18.7 (8)	C44—C45—C46—C41	0.6 (12)
C16—C11—C12—C13	−4.6 (11)	C42—C41—C46—C45	−0.3 (11)
P1—C11—C12—C13	−171.1 (6)	P2—C41—C46—C45	179.5 (6)
C11—C12—C13—C14	3.2 (12)	C43—C44—N2—C3	−14.1 (11)
C12—C13—C14—N1	179.0 (7)	C45—C44—N2—C3	169.9 (7)
C12—C13—C14—C15	−1.6 (11)	C43—C44—N2—C4	−175.5 (7)
N1—C14—C15—C16	−178.8 (7)	C45—C44—N2—C4	8.5 (11)
C13—C14—C15—C16	1.9 (11)	C41—P2—C51—C52	29.2 (7)
C14—C15—C16—C11	−3.7 (12)	C61—P2—C51—C52	140.5 (6)
C12—C11—C16—C15	4.9 (11)	Pt—P2—C51—C52	−99.8 (6)
P1—C11—C16—C15	171.4 (6)	C41—P2—C51—C56	−152.4 (6)
C15—C14—N1—C1	167.2 (7)	C61—P2—C51—C56	−41.1 (7)
C13—C14—N1—C1	−13.4 (11)	Pt—P2—C51—C56	78.6 (6)
C15—C14—N1—C2	8.8 (11)	C56—C51—C52—C53	−1.9 (11)
C13—C14—N1—C2	−171.8 (7)	P2—C51—C52—C53	176.5 (6)
C11—P1—C21—C26	69.3 (7)	C51—C52—C53—C54	0.1 (11)
C31—P1—C21—C26	−176.9 (7)	C52—C53—C54—C55	1.5 (12)
Pt—P1—C21—C26	−55.7 (7)	C53—C54—C55—C56	−1.1 (12)
C11—P1—C21—C22	−107.7 (7)	C54—C55—C56—C51	−0.8 (11)
C31—P1—C21—C22	6.2 (8)	C52—C51—C56—C55	2.3 (11)
Pt—P1—C21—C22	127.3 (6)	P2—C51—C56—C55	−176.1 (6)
C26—C21—C22—C23	−1.3 (12)	C41—P2—C61—C62	23.9 (7)
P1—C21—C22—C23	175.6 (6)	C51—P2—C61—C62	−86.0 (6)
C21—C22—C23—C24	−1.0 (13)	Pt—P2—C61—C62	147.3 (6)
C22—C23—C24—C25	2.3 (13)	C41—P2—C61—C66	−156.6 (6)
C23—C24—C25—C26	−1.3 (13)	C51—P2—C61—C66	93.5 (7)
C22—C21—C26—C25	2.4 (13)	Pt—P2—C61—C66	−33.2 (7)
P1—C21—C26—C25	−174.7 (6)	C66—C61—C62—C63	0.2 (11)
C24—C25—C26—C21	−1.1 (13)	P2—C61—C62—C63	179.7 (6)
C11—P1—C31—C32	−5.3 (8)	C61—C62—C63—C64	1.2 (12)
C21—P1—C31—C32	−110.6 (7)	C62—C63—C64—C65	−1.6 (13)
Pt—P1—C31—C32	125.7 (6)	C63—C64—C65—C66	0.5 (13)
C11—P1—C31—C36	174.5 (6)	C64—C65—C66—C61	1.0 (13)
C21—P1—C31—C36	69.3 (6)	C62—C61—C66—C65	−1.3 (12)
Pt—P1—C31—C36	−54.5 (6)	P2—C61—C66—C65	179.2 (7)
C36—C31—C32—C33	−0.3 (11)	C7—O2—C6—O1	1.1 (13)
P1—C31—C32—C33	179.5 (6)	C7—O2—C6—C5	−177.0 (8)
C31—C32—C33—C34	−1.2 (12)	C6—O2—C7—C8	−179.6 (7)
C32—C33—C34—C35	1.8 (13)		

### Hydrogen-bond geometry (Å, °)

**Table:**

*D*—H···*A*	*D*—H	H···*A*	*D*···*A*	*D*—H···*A*
C5—H5B···Cl2^i^	0.98	2.79	3.601 (10)	141
C64—H64···O1^ii^	0.95	2.39	3.317 (11)	166

Symmetry codes: (i) −*x*+1/2, *y*−1/2, −*z*+1/2; (ii) *x*+1/2, −*y*+1/2, *z*−1/2.
